# Two-Step Transfer Learning Improves Deep Learning–Based Drug Response Prediction in Small Datasets: A Case Study of Glioblastoma

**DOI:** 10.1177/11779322241301507

**Published:** 2025-01-03

**Authors:** Jie Ju, Ioannis Ntafoulis, Michelle Klein, Marcel JT Reinders, Martine Lamfers, Andrew P Stubbs, Yunlei Li

**Affiliations:** 1 Department of Pathology & Clinical Bioinformatics, Erasmus MC Cancer Institute, University Medical Center Rotterdam, Rotterdam, The Netherlands; 2 Department of Neurosurgery, Brain Tumor Center, Erasmus MC Cancer Institute, University Medical Center Rotterdam, Rotterdam, The Netherlands; 3 The Delft Bioinformatics Lab, Delft University of Technology, Delft, The Netherlands

**Keywords:** Transfer learning, deep learning, drug response prediction, glioblastoma

## Abstract

While deep learning (DL) is used in patients’ outcome predictions, the insufficiency of patient samples limits the accuracy. In this study, we investigated how transfer learning (TL) alleviates the small sample size problem. A 2-step TL framework was constructed for a difficult task: predicting the response of the drug temozolomide (TMZ) in glioblastoma (GBM) cell cultures. The GBM is aggressive, and most patients do not benefit from the only approved chemotherapeutic agent TMZ. O6-methylguanine-DNA methyltransferase (MGMT) promoter methylation status is the only biomarker for TMZ responsiveness but has shown limited predictive power. The 2-step TL framework was built on 3 datasets: (1) the subset of the Genomics of Drug Sensitivity in Cancer (GDSC) dataset, including miscellaneous cell cultures treated by TMZ, cyclophosphamide, bortezomib, and oxaliplatin, as the source dataset; (2) the Human Glioblastoma Cell Culture (HGCC) dataset, for fine-tuning; and (3) a small target dataset GSE232173, for validation. The latter two included specifically TMZ-treated GBM cell cultures. The DL models were pretrained on the cell cultures treated by each of the 4 drugs from GDSC, respectively. Then, the DL models were refined on HGCC, where the best source drug was identified. Finally, the DL model was validated on GSE232173. Using 2-step TL with pretraining on oxaliplatin was not only superior to those without TL and with 1-step TL but also better than 3 benchmark methods, including MGMT. The oxaliplatin-based TL improved the performance probably by increasing the weights of cell cycle-related genes, which relates to the TMZ response processes. Our findings support the potential of oxaliplatin being an alternative therapy for patients with GBM and TL facilitating drug repurposing research. We recommend that following our methodology, using mixed cancers and a related drug as the source and then fine-tuning the model with the target cancer and the target drug will enhance drug response prediction.

## Introduction

Cancer is one of the leading causes of death worldwide.^1^ One of the biggest challenges in cancer treatment is tumor heterogeneity; diverse intertumoral genetics lead to distinct treatment responses in patients.^2^ To improve the response rate of therapies and minimize side effects, making accurate predictions of patients’ therapeutic response is a necessity for personalized clinical treatment planning.

Molecular profiling in patient-derived cell cultures, which preserves the molecular characteristics of the parental tumors, has been widely used in preclinical research in the past decades to enable and accelerate cancer therapy development and precision medicine.^3^ Machine learning models have been demonstrated to manage high-dimensional and ever-growing molecular data used for clinical outcome predictions. Particularly, there is a great interest in deep learning (DL) models that can capture the nonlinear interactions between a massive number of features. They are broadly applied to large-scale omics data to facilitate personal diagnosis and prognosis, as well as to reveal insights into cancer mechanisms, exploring biological patterns and cellular processes.^4^ For example, Chiu et al^5^ used DL-encoded expression profiles for response prediction of 265 drugs in 33 cancers and identified drug response modulating oncogenic features. Zhang et al^6^ used gene expression and copy number variation on cell cultures to build interpretable DL models for drug response prediction in cancers. However, the remarkable performance of DL models was based on cancers with large numbers of cell cultures available for training. Ideally, the number of samples should be at least 10 times the number of the parameters to ensure fitness in the DL model training process.^7^ However, for some cancer types, a very limited number of patient samples are available for research purposes, while the heterogeneity and variability of cancers make outcome predictions challenging based on this limited number of samples.^7^ The need for a large training dataset, therefore, restricts the broad application of DL approaches in cancer research.

Transfer learning (TL) is a technique that solves one task by applying the knowledge learned from another (analogous) task to mitigate the small sample size problem of DL models. Specifically, DL models are pretrained on a large source dataset to gain knowledge that then is transferred to a smaller target dataset to enhance the performance on the target dataset. In computer vision, the ImageNet^8^ database has been created to enable the pretraining of TL in image analysis studies. It has been used to improve DL model performance in applications such as automated gender recognition.^9^ TL also has proven its value in the medical research field. Cheerla and Gevaert^10^ have demonstrated improved survival predictions on a small dataset of a specific cancer type when pretraining a DL model on large pan-cancer data. Furthermore, TL has the potential to help with therapeutics development by evaluating the possibility of applying an existing drug in a cancer treatment case that has not yet been tested before.^11^

Given the reported advantages of TL, we decided to exploit DL models together with TL to alleviate the limited training sample problem in drug response prediction. We investigated the optimal way of implementing TL, including exploring different source datasets, and assessing whether refinement in the target domain is beneficial for TL. We demonstrate the added value of TL in a use case which has always been a challenging prediction task in previous studies,^12,13^ namely, predicting temozolomide (TMZ) response in glioblastoma (GBM) patient-derived cell cultures. The GBM is the most aggressive type of brain cancer, but also one of the most lethal cancers with a 5-year survival rate of less than 10%.^14^ It has a low incidence among all cancer types (0.001%) and is therefore considered a rare cancer. TMZ is the only FDA-approved drug since 1999 as the first-line treatment for patients with GBM in combination with radiation therapy, even though one in 2 patients with GBM does not respond to TMZ treatment and still suffer from side effects, such as severe nausea and vomiting.^15^ To date, the methylation status of the MGMT (O6-methylguanine-DNA methyltransferase) promoter is the only well-established biomarker of response for alkylating agents including TMZ.^16^ However, MGMT has shown limited predictive power to distinguish sensitive and resistant patients.^17,18^ Recently, Ntafoulis et al^19^ have shown in a retrospective clinical study that using an ex vivo drug sensitivity screening can predict TMZ response more accurately than MGMT. To further improve the prediction accuracy and minimize the side effects of TMZ, there is a need to develop computational models that can use molecular profiling data generated from functional tumor cell screening projects.

In this study, we constructed a DL framework with a 2-step TL strategy in which knowledge from a large source dataset is first transferred to a more domain-specific dataset in which the model is refined (step 1) and consequently transferred to the (small) target dataset in which a further fine-tuning takes place (step 2). We applied this 2-step TL framework to RNA-seq data of cell cultures derived from treatment-naïve IDH-1 wild-type patients with GBM to predict their response to TMZ. We deployed 3 datasets: (1) The Genomics of Drug Sensitivity in Cancer (GDSC)^20^ dataset that was used as the source dataset, (2) the Human Glioblastoma Cell Culture (HGCC)^21^ dataset that was used as the domain-specific dataset, and (3) a small but well-defined GBM screening dataset GSE232173^19^ that was the final target dataset for which we aimed to predict the TMZ response. We investigated whether and how 2-step TL improves prediction accuracy on the small target dataset. Furthermore, we demonstrated the value of transferring knowledge from other cell cultures and selected drugs, which provides oncologists and pharmacologists with new insights into alternative therapeutic options. The performance of the 2-step TL was extensively compared with 5 categories of benchmark methods: (1) DL models without TL, (2) DL models with 1-step TL, (3) Elastic Net,^22^ (4) a DL framework developed by Theodore Sakellaropoulos et al,^23^ and (5) the predictions based on the expression and methylation status of MGMT.

## Methods

**Figure 1.**
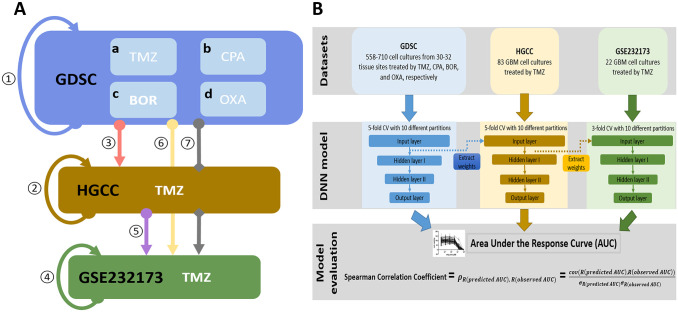
Study design and framework architecture. (A) Study design. The entire study design consists of 7 experiments. *Experiment 1*: Constructing 4 DL models on the GDSC dataset containing mRNA expression of cell cultures from various tissue sites for response prediction of 4 drugs: (a) temozolomide (TMZ), (b) cyclophosphamide (CPA), (c) bortezomib (BOR), and (d) oxaliplatin (OXA). *Experiment 2*: Constructing a DL model on the HGCC dataset containing mRNA expression data of 83 GBM cell cultures for TMZ response prediction. *Experiment 3*: One-step transfer learning (TL) from the DL models of the 4 drugs from the source GDSC dataset to the HGCC dataset and determine the best source drug dataset for the TMZ-treated GBM cell cultures response prediction. *Experiment 4*: Constructing a DL model solely on the GSE232173 dataset to predict TMZ response. *Experiment 5*: One-step TL from HGCC dataset to the target GSE232173 dataset. *Experiment 6*: One-step TL from the source GDSC dataset to the target GSE232173 dataset. *Experiment 7*: Two-step TL from the GDSC source dataset to the GSE232173 target dataset, with the refinement on the HGCC dataset. (B) Experiment settings and framework architecture. The experiments included 3 parts: DL model constructions on the GDSC, HGCC, and GSE232173 datasets. For each part, a 5- or 3-fold CV with 10 repetitions was performed to assess the robustness of the DL models. The transferring process occurred from the source to the target dataset for a particular step at hand. Each time the TL was realized by extracting the weights of the input layer of the DL model trained on the source dataset to initialize the corresponding weights in the target dataset which were subsequently updated during the training. The DL model performance was assessed by Spearman correlation coefficients, where R denotes the ranking of the (predicted or observed) AUC, and cov is the covariance of the ranked variables.

We propose a 2-step TL framework to improve the performance of DL models on small datasets (Figure 1A). The framework consists of 3 parts: First, DL models were pretrained on the source dataset (GDSC) containing miscellaneous cell cultures with response outcomes for multiple drugs. Second, the DL models were then refined on a domain-specific dataset (HGCC), and the best source drug for the transferring task was selected. Finally, the refined DL model based on the dataset of the best source drug was transferred to the final target set (GSE232173) in which the DL model was further fine-tuned. We evaluated the performance of the 2-step TL in comparison with other clinical and computational approaches. The 3 RNA profiling datasets used in the experiment are summarized in Table 1 and detailed in the following section.

**Table 1.** Overview of the treatment-naïve cell culture RNA profiling datasets.

Dataset name	Data type	Sites of tissue	Drug	No. of cell cultures	No. of features
GDSC	Microarray	Various tumor types including GBM	TMZ	861	17 419
			CPA	558	17 419
			BOR	562	17 419
			OXA	710	17 419
HGCC	Microarray	GBM	TMZ	83	23 832
GSE232173	RNA sequencing	GBM	TMZ	22	20 077

Abbreviations: BOR, bortezomib; CPA, cyclophosphamide; GDSC, Genomics of Drug Sensitivity in Cancer; HGCC, human glioblastoma cell culture; OXA, oxaliplatin; TMZ, temozolomide.

More specifically, the GDSC dataset containing cell cultures from various tissue sites treated by 4 drugs was used to pretrain 4 corresponding DL models. The 4 drugs, namely, TMZ, cyclophosphamide (CPA), bortezomib (BOR), and oxaliplatin (OXA), were selected based on their mechanisms of action (MOAs) and availability in the dataset. The 4 trained DL models were transferred and evaluated on TMZ-treated GBM cell cultures from the HGCC dataset to search for the best pretrained DL model that improves TMZ prediction in GBM cultures. This best pretrained model was then refined on the HGCC dataset by training it on TMZ responses in GBM cultures. Finally, this refined DL model was transferred to the target dataset GSE232173 in which the DL model was further refined to predict TMZ response on this small target set of GBM patient-derived cell cultures. The entire study design is shown in Figure 1A.

The drug response on cell cultures was measured and represented by the area under the response curve (AUC).^24^ Specifically, when the drug responsiveness was tested in the cell cultures, the cell viability was measured at discrete points of drug doses, and the AUC was calculated as the definite integral of the cell viability in the dose ranges of interest. The performance of the DL models was evaluated by the Spearman correlation coefficients (SCC):

$$SCC=\rho_{\begin{array}{l} R\left(predicted\;\;AUC\right),\;\;\\ R\left(observed\;\;AUC\right) \end{array}}=\frac{cov\left(\begin{array}{l} R\left(predicted\;\;AUC\right),\;\;\\ R\left(observed\;\;AUC\right) \end{array}\right)}{\begin{array}{l} \sigma_{R\left(predicted\;\;AUC\right)}\\ \sigma_{R\left(observed\;\;AUC\right)} \end{array}}$$

*R* denotes the ranking of the (predicted or observed) AUC, and *cov* is the covariance of the ranked variables.

The metric SCC is nonparametric (ie, without assuming the normal distribution) and determines the monotonic relationship between the rankings of the observed drug AUC and predicted AUC across different cross-validations (CVs) of the datasets, which is more robust to outliers. The CVs of each model were performed 10 times with different CV partitions to test the CV robustness of the models. Since multiple approaches were investigated, we kept the stratified partitions the same for each dataset and each experiment, to ensure a fair comparison of the model performances. The means and standard deviations (SD) of the 10 repetitions were calculated. Figure 1B illustrates our 2-step TL framework architecture and experiment settings.

### Datasets

The datasets GDSC and HGCC were downloaded from their websites. The data generated in the EMC is available on GEO under the number GSE232173.

*GDSC dataset.* The GDSC contains miscellaneous cell cultures from various tumor types treated by multiple drugs. The collection of drugs with distinct MOAs provides an opportunity to test the potential for drug repurposing possibilities. Given the MOA and data availability, TMZ and 3 other drugs CPA, BOR, and OXA were shortlisted for the experiment to determine the source drug dataset for TL. For the 4 drugs, the total number of cell cultures used was shown in Table 1, among which 18 to 34 cell cultures were from GBM tumor tissues, and the rest cell cultures were derived from 29 to 31 other tissue types. TMZ, CPA, and OXA are closely related DNA-damaging agents with chemical structures of triazines, nitrogen mustards, and metal salts drug families, respectively, while TMZ and CPA are both alkylating.^20,25^ The TMZ causes DNA damage that starts cycles of futile reparation and eventually leads to cell death.^25,26^ The CPA,^27^ with a similar MOA to TMZ, crosslinks the strands of DNA and RNA and inhibits transcription synthesis. The OXA binds preferentially to the guanine and cytosine bases in DNA, leading to DNA cross-linking and inhibiting DNA synthesis and transcription.^28^ The BOR, as a proteasome inhibitor, is related to protein stability and degradation pathways and thus serves as a benchmark source drug with the least similar MOA to TMZ among the 4 drugs.^27^

The RNA microarray data in GDSC contains 17 419 features at the gene level. The data were preprocessed with RMA normalization and log2 transformation before usage. Furthermore, given the diverse ranges of the AUC response outcome of the 4 drugs (Supplementary Fig. 1A-1D), the AUC of each drug was min-max normalized into the range of 0 to 10. To prevent overfitting the DL models, feature reduction was performed by filtering out low-variance genes, ie, genes with a variance lower than the 25% quantile of all genes’ variances were removed, which left us with 10 232 genes.

*HGCC dataset*. The HGCC contains RNA data of 83 GBM patient-derived cell cultures before the drug screening measured with a microarray. The dataset was RMA normalized and annotated with 23 832 genes. Log2 transformation was performed before usage. The maximum value of the AUC response was min-max normalized into the range of 0 to 10. The 9955 overlapping genes between GDSC and HGCC were kept for the DL model constructions on the HGCC dataset to enable the TL.

*GSE232173 dataset*. At Erasmus Medical Center, we have built a biobank containing >400 patient-derived GBM cell cultures that maintain the genetic characteristics of the parental tumors.^19,29,30^ The dataset GSE232173 contains RNA sequencing (RNA-seq) data of 22 cell cultures derived from patients with treatment-naïve GBM. First of all, the batch effect was corrected between the first batch of 19 cell cultures and the other 3 cell cultures in the second batch with R package “sva.”^31^ Among the original 20 076 genes, those with low expressions across the 22 cell cultures were removed with the function FilterByExpr in edgeR.^32^ The RNA-seq raw count was normalized with a trimmed mean of M values (TMM), which estimates the effective library sizes, and log2 transformed before usage, with the “CPM” function of R package edgeR.^32^ The AUC response was min-max normalized into the range of 0 to 10. The DL models on GSE232173 were constructed on the 9266 overlapping features between GSE232173, HGCC, and GDSC.

### Framework architecture

The 2-step TL framework was implemented in Python with libraries from Pandas (version 1.1.2),^33^ Numpy (version 1.19.5),^34^ sklearn (version 0.23.0),^35^ TensorFlow (version 1.15.0),^36^ and Keras (version 2.2.4).^37^ The Python code, as well as the weights saved from all the fine-tuned models presented in the article, is available on GitHub (https://github.com/ErasmusMC-Bioinformatics/two-step-TL).

#### DL model without TL

Deep artificial neural network (DNN) models were constructed to predict drug response outcomes, in terms of AUC, on the RNA profiling of cell cultures from GDSC, HGCC, and GSE232173, independently. On GDSC, 4 models were constructed to predict the response to TMZ, CPA, BOR, and OXA for cell cultures from various tumor types, respectively (Figure 1A, Experiment 1). On HGCC and GSE232173, the response to TMZ was predicted for GBM cell cultures (Figure 1A, Experiments 2 and 4). To assess the robustness of the performance of the DL models, a 5-fold CV was performed on GDSC and HGCC and a 3-fold CV was performed on GSE232173 (due to its smaller sample size). This CV was repeated 10 times with different partitions. The input data in the training and test set were normalized separately with StandardScaler to unify the median and the quantile range per feature. The DNN models contained an input layer with genes being the input and an output layer that predicts the normalized AUC value (between 0 and 10) of each cell line. We adopted an architecture of 2 hidden layers, one with 1000 neurons and one with 100 neurons. The weights were initialized using a RandomUniform distribution. The activation functions after the input layer, the first hidden layer, and the second hidden layer are “sigmoid,” “softplus,” and “softplus.” The hyper-parameters were optimized with the 5-fold CV on GDSC, and they were subsequently applied to all DNN models: the dropout rates after the first and the second hidden layers were 0.3 and 0.1, both the regularizations of the activation function kernel and bias were 0.0001, and the loss function was the mean squared error (MSE). The detailed settings of the hyper-parameter tuning and computational resources are shown in Supplementary Methods.^36,38^

#### One-step TL

One-step TL was applied to the 4 DL models described above from the source dataset GDSC to HGCC (Figure 1A, Experiment 3), and the best source drug dataset for TMZ prediction in HGCC was chosen for the subsequent experiments. Specifically, the 4 DL models were first pretrained on the source dataset GDSC with the settings in Experiment 1. The weights of the input layers of the GDSC-based DL models were used to initialize their equivalents in the DL model trained on HGCC. The source drug dataset that resulted in the best 5-fold CV performance on HGCC was selected for further investigation (Figure 1B).

Two more combinations of source and target datasets were tested for the 1-step TL: transferring from HGCC to GSE232173 (Figure 1A, Experiment 5) and transferring from GDSC to GSE232173 (Figure 1A, Experiment 6). These 2 experiments were performed to assess the contribution of GDSC and HGCC in 1-step TL, ie, whether the larger and more diverse GDSC dataset was more informative for TL than the more specific HGCC dataset that contains only GBM cultures. In Experiment 5, the weights of the input layer of the DL model pretrained on HGCC were used to initialize the DL model on GSE232173. In Experiment 6, the weights of the input layer of the DL model pretrained on the best DL model in Experiment 3 (on GDSC) were used to initialize the input layer of the DL model on GSE232173.

#### Two-step TL

We constructed the 2-step TL (Figure 1A, Experiment 7) extending the 1-step TL (Figure 1A, Experiment 3) to improve the prediction on the small dataset GSE232173. Similarly, the weights of the input layer of the model with the best performance on HGCC in Experiment 3 were extracted to initialize the DL model on the GSE232173 and a 3-fold CV was carried out and repeated 10 times to make the TMZ response prediction on dataset GSE232173 (Figure 1B). By comparing Experiment 7 with Experiment 6, we evaluated whether the extra refinement on HGCC, which represented the target domain, helped with the final prediction on GSE232173.

### Benchmark methods

To assess the performance of the 2-step TL framework extensively, we compared its performance to 3 other benchmarks. Next to a statistical test on MGMT methylation status, all other experiments using machine learning methods were performed on GSE232173 with a 3-fold CV and repeated 10 times using the same seeds in all experiments (including the DL models).

First, we assessed the clinical relevance of our framework: ie, a correlation analysis between the MGMT methylation status (ie, methylated or unmethylated) and AUC outcomes of the TMZ-treated GBM cell cultures was performed using the Wilcoxon signed-rank test. This indicated the predictive power of the current clinical biomarker of TMZ response in patients with GBM on GSE232173. Furthermore, we fitted a polynomial regression model (degree = 2) using the MGMT expression as the single predictor and compared its performance with our framework.

Second, we compared the performance of our framework with that of an Elastic Net (EN) model, which is widely used in drug response prediction.^12,39^ Feature selection was performed before the EN model construction on the training set of each CV, where the top 5, 10, 15, 20, 50, and 100 features based on F-statistics were used to construct the model, respectively.

Third, we employed the DL model from the study of Sakellaropoulos et al,^23^ where a DL model was constructed based on highly varied RNA expression genes to predict the outcomes of drug response. For this comparison, we implemented the DL model using the original code on the GSE232173 dataset, including the hyper-parameter optimization.

## Results

### Without TL

**Figure 2.**
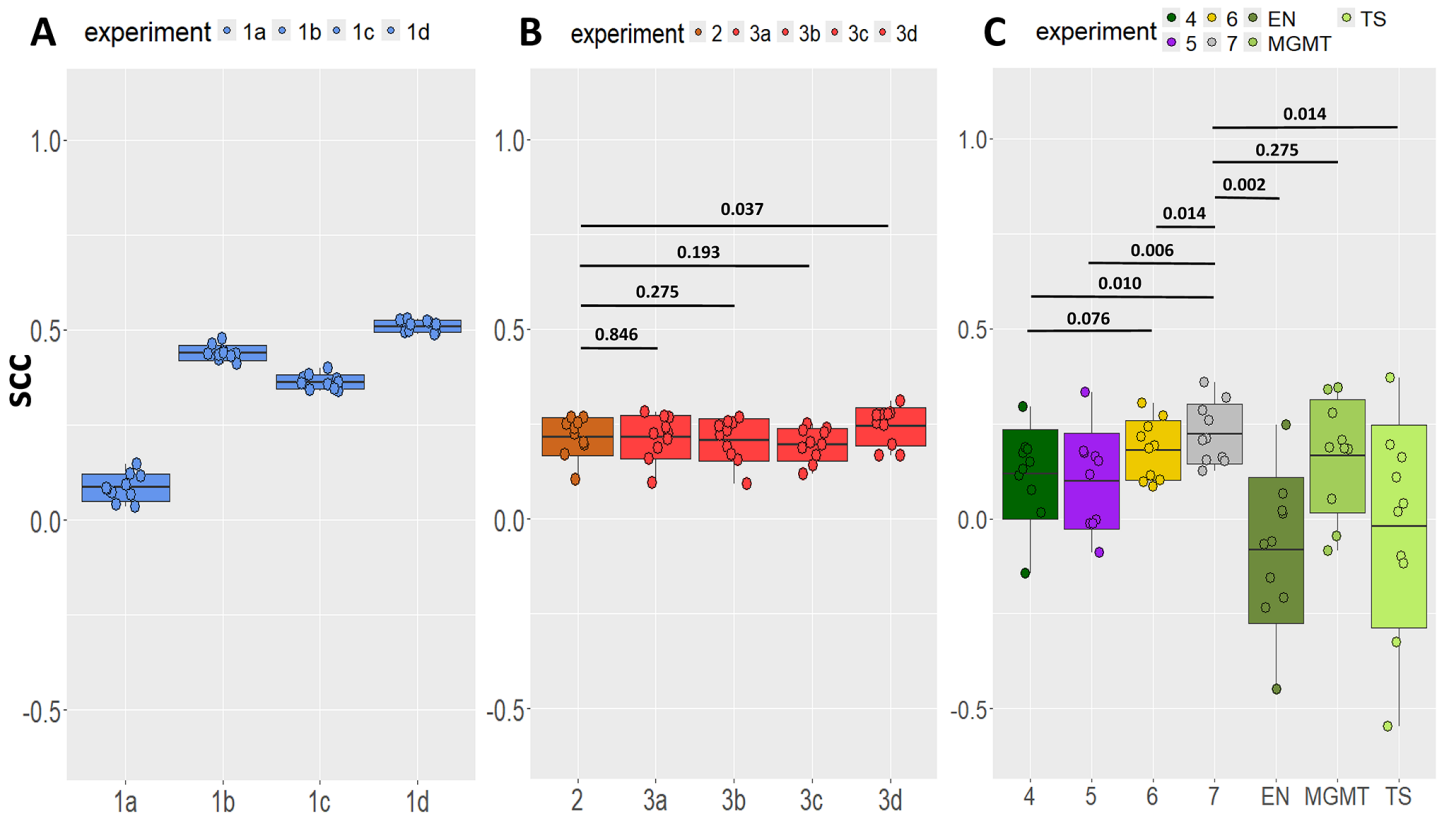
The performance of the DL models with 10-times cross-validations with different partitions. In the boxplots, the mean Spearman correlation coefficients (SCC) of the cross-validations with standard deviations are shown for the 10 experiments. (A) Experiments on GDSC without TL for the response prediction of 4 drugs. Experiment 1a: TMZ, Experiment 1b: CPA, Experiment 1c: BOR, and Experiment 1d: OXA. (B) Experiments on HGCC to predict the response to TMZ. Experiment 2: without TL. Experiments 3a-3d: with 1-step TL from GDSC using source drugs TMZ, CPA, BOR, and OXA, respectively. The significance of the performance difference is shown in *P* values. (C) Experiments on GSE232173 to predict the response to TMZ. Experiment 4: without TL. Experiments 5 and 6: with 1-step TL from HGCC TMZ and GDSC OXA, respectively. Experiment 7: with 2-step TL from GDSC OXA and refined on HGCC TMZ. Experiment MGMT: predictions made by a polynomial regression using the expression of gene MGMT as the predictor. Experiment EN: predictions made by an Elastic Net model. Experiment TS: predictions made by a DL model constructed by Theodore Sakellaropoulos et al. The significance of the performance difference between Experiment 7 and the others was shown in *P* values.

On GDSC (Figure 2A, Experiments 1a-1d), the OXA-based DL model gave the best prediction performance with an average SCC of 0.509 (SD 0.015), followed by CPA with an SCC of 0.439 (SD 0.019), BOR with an SCC of 0.363 (SD 0.019), and TMZ with an SCC of 0.085 (SD 0.035). These results showed that the DL models could predict OXA, BOR, and CPA response on the GDSC dataset with relatively high accuracy, while the response to the drug TMZ was close to impossible to predict. To inspect the influence of outliers on the performance, we made Q-Q plots: CPA, BOR, and OXA all were roughly normally distributed (Supplementary Fig. 2B-2D), TMZ, on the contrary, was more skewed, and therefore more difficult to predict GDSC (Supplementary Fig. 2A).

On HGCC, the DL model with a 5-fold CV (Figure 2B, Experiment 2) to predict GBM cell cultures’ response to TMZ resulted in an average SCC of 0.217 (SD 0.052), which is better than the result in the larger GDSC dataset (Experiment 1a). This might be because around 90% of the GDSC TMZ-treated cell cultures were very resistant (original AUC > 0.95) to this drug while the AUC distribution of HGCC was more balanced (Supplementary Fig. 1A and 1E), which is confirmed by its Q-Q plots (Supplementary Fig. 2A and 2E).

On GSE232173 (Figure 2C, Experiment 4), the DL model without TL resulted in an average SCC of 0.120 (SD 0.118) for GBM cell culture TMZ response prediction. As expected, this is worse than the result on HGCC (Experiment 2) which is relatively a larger dataset. Interestingly, it is better than the result on the largest dataset GDSC (Experiment 1a). Not only the histograms of the AUC of the 3 datasets (Supplementary Fig.1A, 1E, and 1F) but also the Q-Q plots (Supplementary Fig. 2A, 2E, and 2F), suggested a more balanced distribution of TMZ in the GSE232173 dataset than in the GDSC dataset, which seems crucial for DL model construction.

### One-step TL

The best DL model for TL was determined in the 1-step TL from GDSC to HGCC (Figure 1A, Experiment 3), in which we evaluated from which drug the model learned the most from the GDSC dataset to perform TMZ response prediction in HGCC. This was the OXA-based model. Transferring the weights from the OXA-based model on GDSC cell cultures to the DL model on HGCC (Figure 2B, Experiment 3d) achieved an increased SCC of 0.244 (SD 0.050), compared with without TL (Experiment 2). Wilcoxon signed-rank test was performed to compare the 10 SCC pairs from Experiments 2 and 3d, which were obtained from 10 repetitions of 5-fold CV with identical partitions in both experiments, yielding a *P* value of .037, confirming a significant improvement by the transferred model. Transferring from other drugs including TMZ (SCC 0.216), CPA (SCC 0.208), and BOR (SCC 0.196) did not help (Figure 2B, Experiments 3a, 3b, and 3c, respectively). Notably, transferring from BOR gave the lowest performance in agreement with the different MOA of BOR compared with TMZ. The TMZ-based TL did not help the prediction due to the skewed distribution and lack of sensitive cultures in the source dataset (Supplementary Figs. 1A and 2A). Interestingly, CPA, considered the drug with the most similar MOA to TMZ, was not identified as the best source for TL. According to the AUC distribution of CPA on GDSC (Supplementary Figs. 1B and 2B), where cell cultures with AUCs less than 0.9 were absent, this might be due to insufficient information available from the CPA-sensitive cell cultures. Finally, we selected OXA as the best source drug dataset among the 4 to pretrain the DL model for GBM cell culture response prediction to TMZ.

Based on the best source drug dataset OXA in GDSC, the 1-step TL from GDSC to GSE232173 (Figure 2C, Experiment 6) achieved a higher SCC of 0.181 and a smaller SD of 0.078 compared with the results of Experiment 4 (direct training of DL model on GSE232173), although the improvement of the SCC was not significant (*P* = .076). Transferring from TMZ in HGCC to GSE232173 (Figure 2C, Experiment 5) decreased the DL performance with an SCC of 0.103 (SD 0.126). These 2 experiments highlight the importance of having sufficient samples in the pretraining since the dataset of HGCC contains only 83 samples while the GDSC OXA dataset contains 710 samples.

### Two-step TL

After the DL model was pretrained on the GDSC OXA dataset and transferred to HGCC in the 1-step TL, the model was extended further to 2-step TL by training the model on HGCC (ie, refining) before being transferred to and validated on GSE232173. During the validation on GSE232173, this 2-step DL model (Figure 2, Experiment 7) achieved an average SCC of 0.222, which was significantly higher than the model trained without TL (Figure 2, Experiment 4, SCC = 0.120, *P* = .010). This demonstrates that the 2-step TL is superior to not using TL at all. The SD (= 0.079) of the 2-step TL is much smaller than the SD without TL, suggesting that the 2-step TL also increased the stability of the DL models. Furthermore, compared with 1-step TL from HGCC and from GDSC to GSE232173 (Figure 2, Experiments 5 and 6d, respectively), 2-step TL succeeded in boosting the performance significantly (*P* values = .006 and .014, respectively). Together, the fact that 2-step TL was able to make a significant improvement indicates both TL steps are important, ie, starting with a large and general source domain and then refinement in the target domain.

### Other benchmark methods

#### Biomarker MGMT

To evaluate the clinical relevance of our 2-step TL framework, we compared it with the performance using the methylation status and expression of the MGMT gene; the most well-studied predictive marker of TMZ response in patients with GBM.^18^ The MGMT promoter methylation status was available for 20 out of the 22 samples in GSE232173. However, the gene expression of MGMT was weakly correlated to the AUC of GSE232173 with an SCC of 0.128 (Supplementary Fig. 3A), and no significant difference was observed between the MGMT-methylated versus nonmethylated groups regarding their responses to TMZ (Wilcoxon rank-sum test *P* = .710, Supplementary Fig. 3B). This demonstrates the insufficiency of using MGMT methylation status or gene expression directly as a biomarker in clinics, and underpins the necessity of our computational models using molecular profiling data generated from functional tumor cell screening project.

When used as the only input variable of a polynomial regression model (degree = 2), the MGMT expression gave a mean SCC performance of 0.167 (±0.148) (Figure 2C, Experiment MGMT). This reasonable performance supports the use of MGMT in clinical practice but with the help of a properly trained machine learning model and not by its methylation status as currently used. However, the model was unstable and its performance suffered from high variance (ie, for some repetitions, the SCCs were close to 0, which was a random prediction), just as the DL models without TL did (in Experiment 4). Thus, we demonstrate that our 2-step TL improved performance as well as prediction stability than the machine learning model based on the expression of MGMT.

#### Elastic Net

The performance of our 2-step TL framework was compared with that of EN. The EN model with the top 10 genes achieved the best performance of an average SCC of −0.082 (SD = 0.192, Figure 2C, Experiment EN), which was not only significantly worse than the 2-step TL (*P* = .002) but also worse than the performance of the DL models on GSE232173 without TL.

#### DL by Sakellaropoulos et al

Numerous studies have worked on drug response predictions using machine learning methods. To assess the robustness of our DL framework, we also compared the performance of our 2-step TL with that of the DL model built by Theodore Sakellaropoulos et al.^23^ This Sakellaropoulos model resulted in an SCC = –0.020 with the largest SD (= 0.266) among all experiments (Figure 2C, Experiment TS), which is significantly worse than the 2-step TL (*P* = .014).

### Biological interpretation

To reveal the biological processes our DL models have learned about TMZ resistance in GBM cultures, we extracted the weights at the input layer from the 3 DL models built on GSE232173: one without TL (Experiment 4), one with 1-step TL from GDSC (Experiment 6), and one with 2-step TL (Experiment 7). Afterward, we performed Gene Set Enrichment Analysis (GSEA)^40^ on these 3 sets of weights for each gene. Specifically, the weights of the genes in each model were used to rank the genes, which served as the input of the GSEA analysis querying against the Hallmark database.^41^ Pathways with a false discovery rate (FDR) lower than 0.25 are reported.

**Figure 3.**
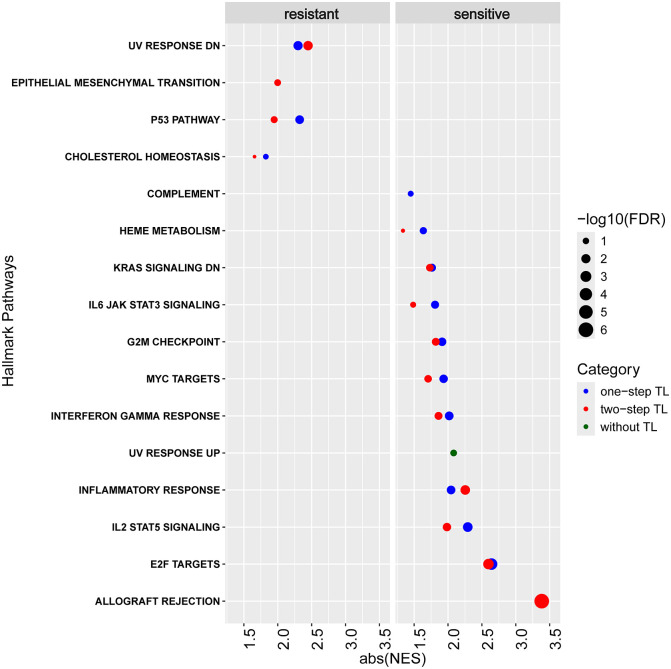
Hallmark pathways identified by Gene Set Enrichment Analysis using the weights of the genes at the input layer of 3 deep learning (DL) models on the GSE232173 dataset. Pathways were identified from the weights of the DL models without transfer learning (TL, Experiment 4, in green), with 1-step TL (Experiment 6, in blue), and with 2-step TL (Experiment 7, in red), ranked by the absolute values of the normalized enrichment scores (abs(NES)). The bigger size of the circles indicates the more significance of the pathways (in terms of the false discovery rates).

The results showed that the TL not only improved the prediction performance but also revealed more knowledge about the GBM drug response mechanisms (Figure 3). Without TL, the only observed pathway was the upregulation of UV response in the sensitive responsiveness. This was also confirmed by the results with both 1-step and 2-step transferring, where the UV response was downregulated in the resistant responsiveness.

Pathways identified based on 1-step and 2-step TL largely overlapped (Figure 3), most of which have been reported to play an important role in carcinogenesis, drug resistance, and immune functions, such as IL2 STAT5 signaling, inflammatory response, and KRAS signaling.^42-44^ More importantly, the cell cycle-related pathways, such as E2F targets, G2M checkpoint, and MYC targets, were observed from the models transferred from OXA in the sensitive group (Supplementary Table 1 and Supplementary Fig. 4). G2M checkpoint and E2F targets regulate the cell cycles and DNA damage repair.^45^ The transcription factor MYC also serves as the TMZ resistance driver.^46^ Besides UV response DN, the p53 pathway and epithelial-mesenchymal transition were enriched in the resistant cell cultures. Both of them have been demonstrated to induce TMZ resistance in GBM.^47,48^

## Discussion

In this study, we explored the added value of TL techniques for the prediction task of drug response in studies with limited samples and showcased the TMZ drug response prediction task in GBM patient-derived cell cultures, which is a difficult prediction task. Multiple DL prediction models were constructed to predict the response to TMZ in a small dataset GSE232173 which only contains 22 samples. Besides DL prediction models with or without TL, we also applied state-of-the-art machine learning prediction models and biomarkers used in the clinic for benchmarking purposes.

We advocate the 2-step TL for the drug response prediction problem using a broad range of mixed cancers and related drugs as the source and fine-tuning the model with the target cancer and target drug. Compared with the DL model without TL, 1-step TL from the OXA dataset in GDSC improved the average prediction accuracy on HGCC and GSE232173 for TMZ response prediction. Furthermore, the 2-step TL, which added the refining step in the target domain after the DL model was pretrained on a broader dataset, facilitated the performance on small target datasets significantly, whereas the 1-step TL did not, which indicated that pretraining should be done on a relatively large dataset. Notably, the variance of the DL models was largely decreased when TL was applied on GSE232173, which suggests that TL improved the stability and robustness of drug response prediction in the small dataset (Supplementary Table 2). Moreover, we tested the DL model performance with a 5-fold CV on GDSC and HGCC and a 3-fold CV on GSE232173, with 10 identical CV partitions for different experiments on each dataset for fair comparisons. In addition to the observation of a significant improvement in the mean SCC scores and decreased SD with 1-step TL on HGCC (Figure 2B, Experiment 3d) and 2-step TL on GSE232173 (Figure 2C, Experiment 7), we inspected the impact of TL on each repetition on HGCC and GSE232173 to ensure the robustness of TL given different partitions. It is shown that 1-step TL on HGCC (Experiment 3d) and 2-step TL on GSE232173 (Experiment 7) consistently achieved higher performance on most repetitions (Supplementary Fig. 5). The CPA-, BOR-, and OXA-based models on GDSC, which is the biggest dataset with miscellaneous cell cultures, achieved relatively higher performance (ie, higher SCC scores) and stability (ie, smaller SD). It demonstrates the capability of DL models to predict the drug response for a mixture of cell cultures from various tumor types. The low performance of the TMZ-based model on datasets GDSC, HGCC, and GSE232173 alone consistently confirms the challenge of TMZ response predictions on cell cultures from various tissue types, including GBM. In this challenging dataset, the clinical biomarker MGMT methylation status failed to show a correlation with TMZ response. Moreover, our framework outperformed an Elastic Net model and another DL framework and showed increased model stability.

The hallmark pathways identified from the OXA-based TL revealed more pathways associated with cell proliferation compared with those without TL. The top 5 most influential genes (with the highest weights) are *RSL1D1* (involved in the regulation of apoptotic process and regulation of cellular senescence), *NPM1* (involved in centrosome duplication, protein chaperoning, and cell proliferation processes), *PHB2* (involved in positive regulation of cell cycle phase transition), *HPRT1* (involved in the generation of purine nucleotides), and *PPM1G* (involved in the encoding of negative regulators of cell stress response).^49^ The finding that the overexpression of these cell proliferation-related genes is associated with a better drug response is consistent with previous studies.^50,51^ Specifically, Chawla et al showed that highly proliferative tumor cells tend to be more sensitive to TMZ in prostate cancer because rapidly dividing cells are actively going through the cell cycle and TMZ induces double-strand breaks in the post-treatment cell cycle.^52^ The 3 cell cycle–related pathways, E2F targets, G2M checkpoint, and MYC targets, together with the UV response, have been identified in our previous study^19^ on the GSE232173 cohort in both patient-derived cell cultures and matching patient tumor tissues. In the previous study, GSEA was performed on the differentially expressed genes between responders and nonresponders (for cell cultures, the responsiveness was defined based on the cell viability, while for tumor tissues, this was defined based on progression-free and overall survival of the patients) of TMZ-treated GBM samples. It is known that OXA and TMZ are both classified in the mechanism of action category: cross-linking/alkylation.^53^ The TMZ induces DNA damage by methylating guanine residues, leading to mismatches during DNA replication, whereas OXA forms DNA crosslinks, which prevent DNA replication and transcription.^54,55^ This means both TMZ and OXA can activate cell death pathways through various mechanisms. The fact that the cell cycle–related pathways were observed only after OXA-based TL inferred that the TL framework increased response prediction performance probably because the transferred weights from OXA-based models put an emphasis on cell cycle–related genes, which are involved in cell death and DNA repair and important for TMZ response. Therefore, we inferred that the model could predict the likelihood of cell death in GBM cells. The functional correlation between the 2 drugs has been observed in the study of Roberts et al where they proved that OXA could induce cell apoptosis and reduce the expression level of MGMT, leading to a more sensitive response to TMZ treatment.^56^

We investigated TL, which transfers the knowledge from a well-trained source model and refines the model with more prediction target-specific data later. As an extra validation, we opted for another approach to ensure the robustness of our methodology, based on GDSC and HGCC databases but with a different definition of the source, fine-tuning, and target set. We used all non-GBM cell lines treated by OXA in GDSC as the source dataset and took the 34 GBM cell cultures from the GDSC treated by TMZ as the fine-tune set to demonstrate the value of our 2-step TL. Subsequently, HGCC became the independent target set for the external validation. The result showed (Supplementary Fig. 6) that the 2-step TL (Experiment 7) boosted the performance of DL significantly (*P* = .027) compared with no TL (Experiment 4). Also, the 2-step TL (Experiment 7) performed better than the 1-step TL in Experiment 5 (*P* = .004). In Experiment 6, where the 1-step TL was performed between the pretraining on GDSC without the fine-tuning step in the target domain of TMZ-treated GBM samples and directly transferred to the target domain (TMZ response in GBM), an improvement of SCC scores was observed with less stability. This again demonstrates the value of our 2-step TL where first general knowledge is learned from a big(ger) source and then it is fine-tuned and focused on the target domain for the final task.

More specifically, we inspected the source dataset used for pretraining. For the TMZ prediction using a DL model without TL (ie, Figure 1A, Experiment 1a), almost 90% of the cell cultures from GDSC were resistant to TMZ with AUC values larger than 0.95 (Supplementary Fig. 1A), which resulted in poor prediction performance when trained on this dataset. In the HGCC and GSE232173 datasets, the AUC distributions were less skewed than in the GDSC dataset (Supplementary Figs. 1E, 1F, 2E, and 2F), and thus, the DL models trained on these datasets achieved much higher SCCs than the one on the GDSC datasets. Furthermore, we observed that the OXA dataset produced the best performance as the source drug dataset; OXA belongs to the same MOA category as TMZ and CPA. The TMZ-treated samples in the GDSC dataset had a very skewed distribution (Supplementary Figs. 1A and 2A), which we believe caused the failure of TMZ response prediction in GDSC. CPA, although with an even more similar MOA to TMZ than OXA, was incapable of improving the performance when used to transfer to HGCC, which was probably caused by the lack of samples with an AUC < 0.9 (ie, responders) in the GDSC dataset (Supplementary Fig. 1B). Notably, for BOR, with the most dissimilar MOA compared with TMZ, the transfer to the TMZ-based model on HGCC gave the worst performance among the 4 drugs. Together, we conclude that in order to transfer models from another dataset, these models should be trained not only on similar drugs (ie, with similar MOA) but also should have seen a balanced group of responders and nonresponders, as well as have to be of a reasonable size. In future studies, new clinical problems in need will be defined as a validation and extension of the current framework.

In addition, we evaluated the impact of different sample sizes of the target datasets on the performance of the 2-step TL framework (Experiment 7). Random sampling was performed without replacement to generate subsets with sample sizes of 10, 13, 15, 18, and 20. The random sampling was repeated 3 times with different sample selections. The experiments were implemented the same way as for the whole GSE232173 with 22 samples, ie, the weights of the first layer of the DL model were the same across different target set sample sizes, which were initialized using those extracted from the pretrained and refined DL models. Also as in Experiment 7, a 3-fold CV was performed on each target dataset, with 10 repetitions with different partitions. For each sample size, the results in 3 random samplings were presented in 3 different colors in the figure below. The mean and standard deviation of the SCC scores per sampling in each subset were calculated and shown in line plots with shadings. We observed from these results that smaller sample sizes of the target set generally result in larger variations in the SCC scores (Supplementary Fig. 7). Notably, the performance of the model became less dependent on the selection of the samples when the sample size increases, ie, at the sample size of 20, the 3 samplings resulted in similar performance. Therefore, we demonstrated that we have sufficient samples in the target set for a fair evaluation of the model performance. This also suggested that the presence of sufficient representative samples (eg, sample size >20 in this analysis) ensures the generalizability and stability of the models. The GBM is a very rare yet heterogeneous cancer, and therefore there is a strong need to implement TL to mitigate the lack of data problem.

To develop a more robust model for TMZ response prediction in the future, more experimental data generated from drug screening efforts would contribute the most. This means more GBM patient-derived cell cultures with balanced TMZ response outcomes (ie, both resistant and sensitive) and testing on a broad range of anticancer drugs could help with the exploration of drug repurposing. Computationally, there are also strategies to learn from other sources to reduce the training burden on limited data by implementing TL or meta-learning. In meta-learning, a model is trained by learning from a variety of different tasks, such that the model has gained generalizable knowledge and could be adapted to new tasks efficiently and flexibly.^57^ Meta-learning has considerable potential in the medical field. The generalizability of the meta-learning models enables one-shot or few-shot learning on individual patient data for personalized medicine and rare disease studies without enough labeled samples.

Regarding the metric to represent the sensitivity of cell cultures in drug screening, we had to use AUC since it is the only metric available in HGCC. However, we believe that having a more standardized metric would greatly enhance transferable information. Area under the response curve is a measure of how much cell viability increases upon drug exposure. Inevitably, there exist some discrepancies in AUC calculation in the 3 datasets in this study. That is, the drug dose range given to the cell cultures differs among the drugs within one cohort as well as between cohorts. Furthermore, the AUC curves were fitted in different manners: GDSC calculated the AUC with a 5-parameter model,^58^ while HGCC used a 4-parameter model.^21^ For GSE232173, the AUC was calculated in GraphPad where the area of the trapezoids under the segments between 2 connecting dose ranges was added.^59^ All the inconsistencies suggest that the AUC values between datasets are not directly comparable. Therefore, the drug response metric AUC was not considered as an absolute response value of cell cultures but was used to rank the relative sensitivity of cell cultures to the drugs. The concordance of the ranking between the predicted and observed drug sensitivity was evaluated by the SCC. While this approach distinguishes the responders and nonresponders in a dataset, there is certainly a need to unify the measurement of drug response AUC across datasets to facilitate the generalizability of drug response studies.

## Conclusions

We developed and implemented a 2-step TL framework and tested its utility in a challenging case of TMZ response prediction on GBM patient-derived cell cultures. We demonstrated that our 2-step TL framework improved the robustness and stability of DL models on a small dataset compared with without and 1-step transferring. Besides the clinical biomarker MGMT methylation status, our model is also superior to 3 other state-of-the-art machine learning methods. The utilization of 2-step TL demonstrates the benefits of pretraining the DL model on a broad dataset with a balanced distribution of prediction classes and the necessity of refining the DL model in the target domain. The 2-step framework could be used in the prediction of other drugs for the treatment of cancers, where a limited number of patient samples is available. Further, this study highlighted the importance of identifying the optimal source drug dataset for the transferring and helped to explore the possibility of drug repurposing: While the current first-line treatment for patients with GBM is the chemotherapy drug TMZ, OXA could be investigated in future research as an alternative treatment for TMZ-resistant patients, given that the antitumor effects of OXA as a treatment for GBM have been indicated in a previous study.^56^ Our findings manifest the potential of TL utilization in new therapy development, and we believe that our exploration and recommendations on TL construction will advance this development.

## Supplemental Material

sj-docx-1-bbi-10.1177_11779322241301507 – Supplemental material for Two-Step Transfer Learning Improves Deep Learning–Based Drug Response Prediction in Small Datasets: A Case Study of Glioblastoma Supplemental material, sj-docx-1-bbi-10.1177_11779322241301507 for Two-Step Transfer Learning Improves Deep Learning–Based Drug Response Prediction in Small Datasets: A Case Study of Glioblastoma by Jie Ju, Ioannis Ntafoulis, Michelle Klein, Marcel JT Reinders, Martine Lamfers, Andrew P Stubbs and Yunlei Li in Bioinformatics and Biology Insights

sj-xlsx-2-bbi-10.1177_11779322241301507 – Supplemental material for Two-Step Transfer Learning Improves Deep Learning–Based Drug Response Prediction in Small Datasets: A Case Study of Glioblastoma Supplemental material, sj-xlsx-2-bbi-10.1177_11779322241301507 for Two-Step Transfer Learning Improves Deep Learning–Based Drug Response Prediction in Small Datasets: A Case Study of Glioblastoma by Jie Ju, Ioannis Ntafoulis, Michelle Klein, Marcel JT Reinders, Martine Lamfers, Andrew P Stubbs and Yunlei Li in Bioinformatics and Biology Insights
