# Supplementary material for: Two-Step Transfer Learning Improves Deep Learning–Based Drug Response Prediction in Small Datasets: A Case Study of Glioblastoma
Source: Bioinform Biol Insights. 2025 Jan 3;19:11779322241301507. doi: 10.1177/11779322241301507 (PMC11700395; doi:10.1177/11779322241301507)
Supplement: sj-docx-1-bbi-10.1177_11779322241301507 – Supplemental material for Two-Step Transfer Learning Improves Deep Learning–Based Drug Response Prediction in Small Datasets: A Case Study of Glioblastoma [file sj-docx-1-bbi-10.1177_11779322241301507.docx]

# Supplementary

**Supplementary Figure 1**. The AUC distribution of drugs **(A)** TMZ on GDSC, **(B)** CPA on GDSC, **(C)** BOR on GDSC, **(D)** OXA on GDSC, **(E)** TMZ on HGCC, **(F)** TMZ on GSE232173.


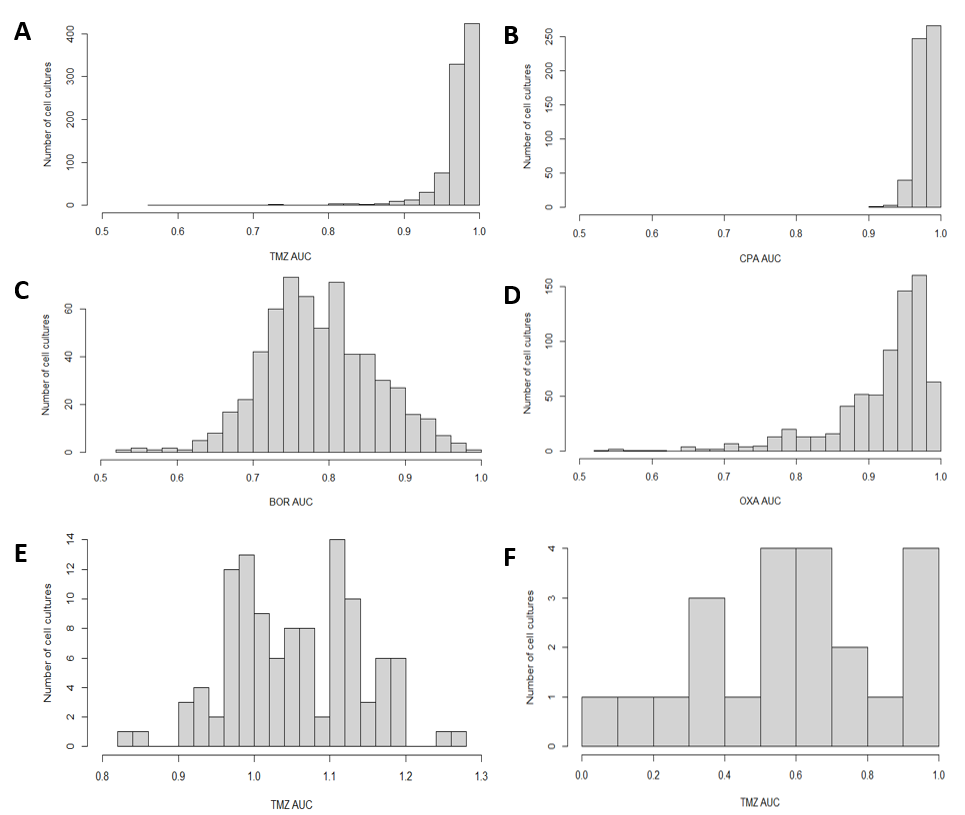


**Supplementary Figure 2**. The Q-Q plots of drugs **(A)** TMZ on GDSC, **(B)** CPA on GDSC, **(C)** BOR on GDSC, **(D)** OXA on GDSC, **(E)** TMZ on HGCC, **(F)** TMZ on GSE232173. The data is nearly a normal distribution when it forms a roughly straight line in the Q-Q plots.


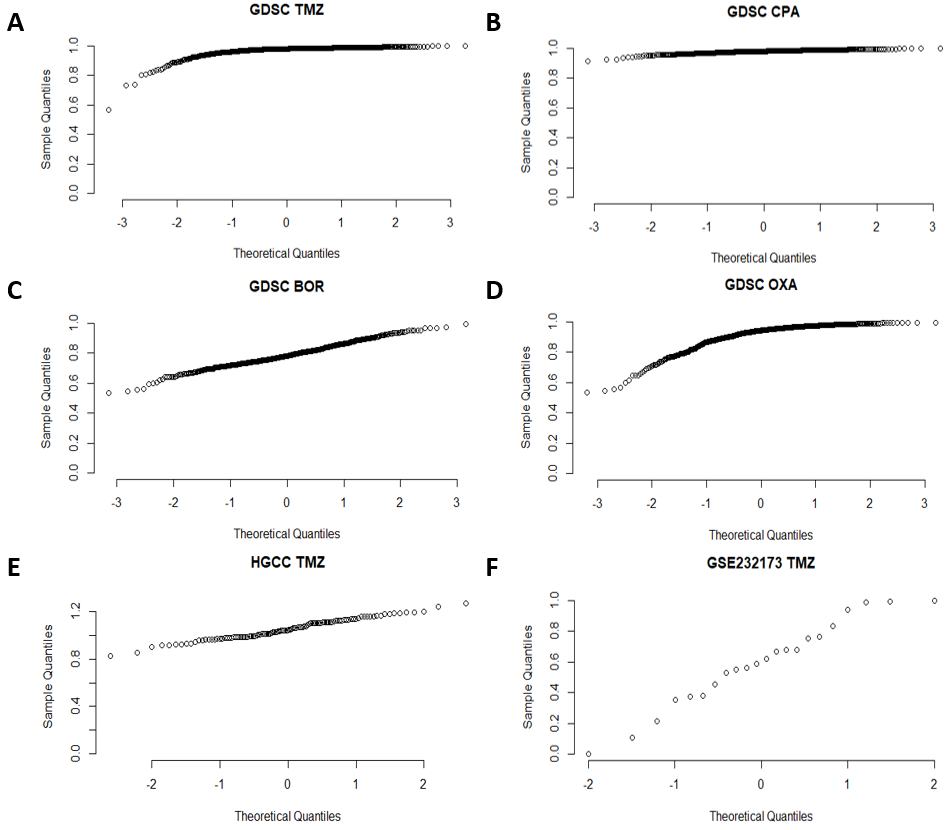


**Supplementary Figure 3**. **(A)** Spearman Correlation between O^6^-methylguanine-DNA methyl-transferase (MGMT) expression values and TMZ AUC outcomes on GSE232173 dataset. **(B)** The boxplot of the TMZ AUC outcomes for the GSE232173 samples grouped by the methylation status of MGMT.


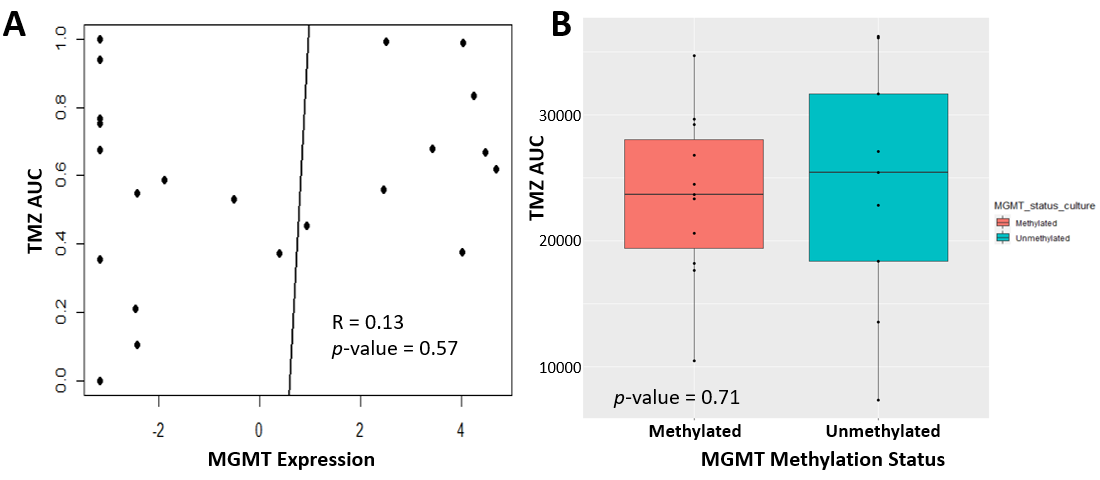


**Supplementary Figure 4.** Cell cycle-related pathways, including MYC targets, E2F Targets and G2M checkpoint. The weights of the gens are normalized to the mean weights of all the genes in the target set. The genes with weights above the average are marked in red, otherwise in grey.


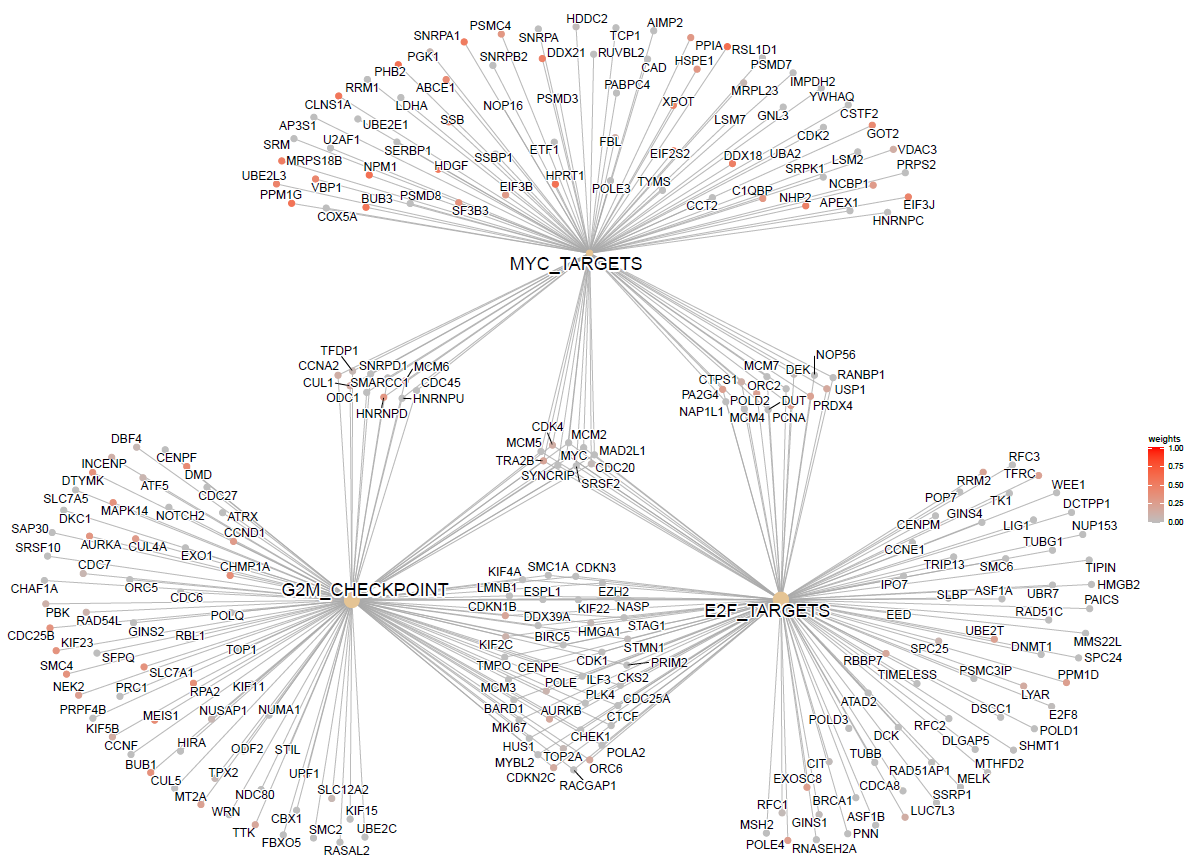


**Supplementary Figure 5**. The results of deep learning models with 10 repetitions **(A)** on HGCC: without (Experiment 2) and with one-step (Experiment 3a-3d) transfer learning and **(B)** on GSE232173: without (Experiment 4), with one-step (Experiment 5 & 6) and with two-step (Experiment 7) transfer learning.


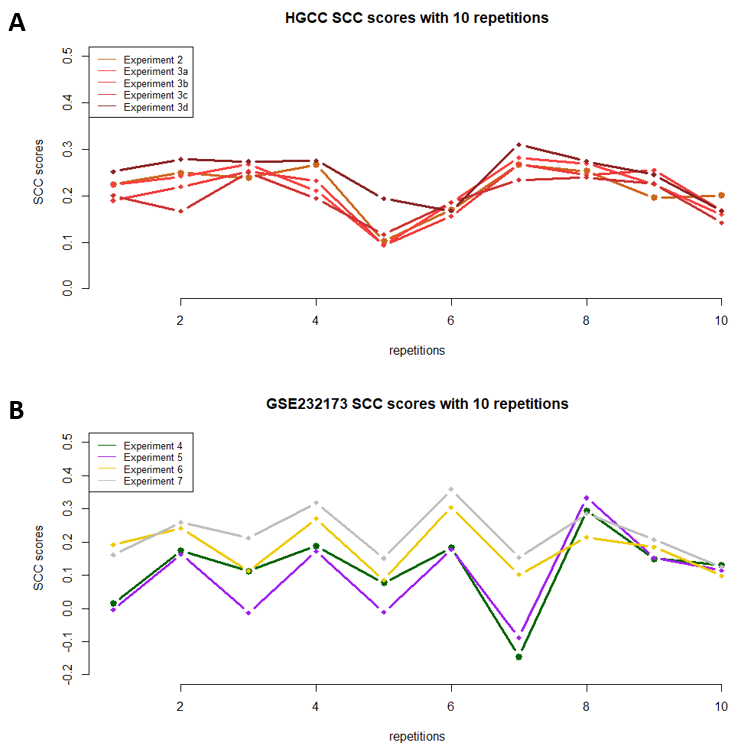


**Supplementary Figure 6.** Evaluating the robustness of our methodology, based on GDSC and HGCC databases but with a different definition of the source, fine-tuning, and target set. All non-GBM cell lines treated by OXA in GDSC as the source dataset, and took the 34 GBM cell cultures from the GDSC treated by TMZ as the fine-tune set to demonstrate the value of our two-step TL. Subsequently, HGCC became the independent target set for the external validation. Experiment 4: without TL. Experiments 5 & 6: with one-step TL from GDSC TMZ-treated GBM and GDSC OXA non-GBM, respectively. Experiment 7: with two-step TL from GDSC OXA non-GBM and refined on GDSC TMZ-treated GBM.
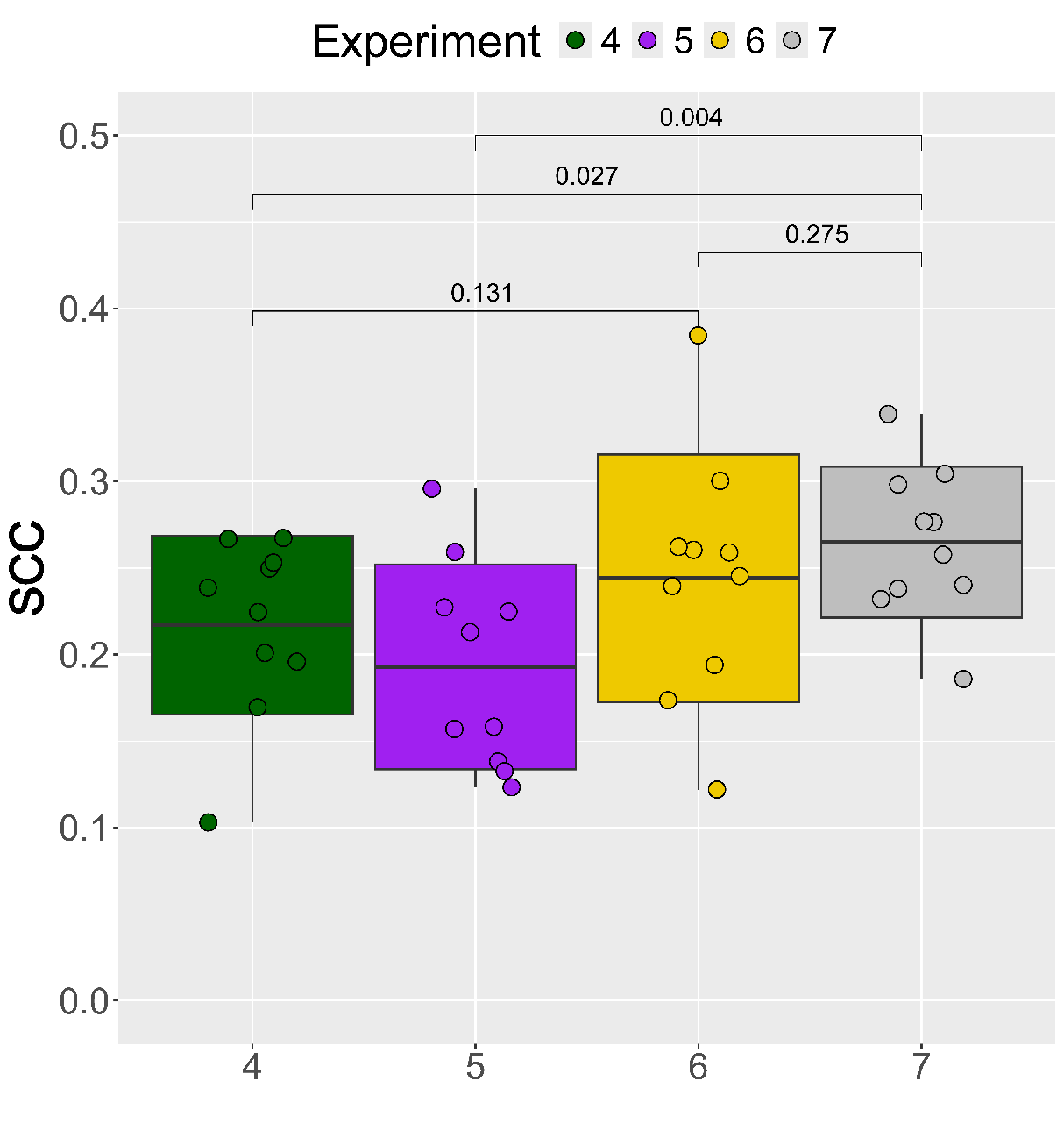


**Supplementary Figure 7.** Impact of different sample sizes of the target set on the performance of the two-step TL framework (Experiment 7). Random sampling without replacement was performed to generate subsets with sample sizes of 10, 13, 15, 18, and 20. The random sampling was repeated three times with different sample selections**.**


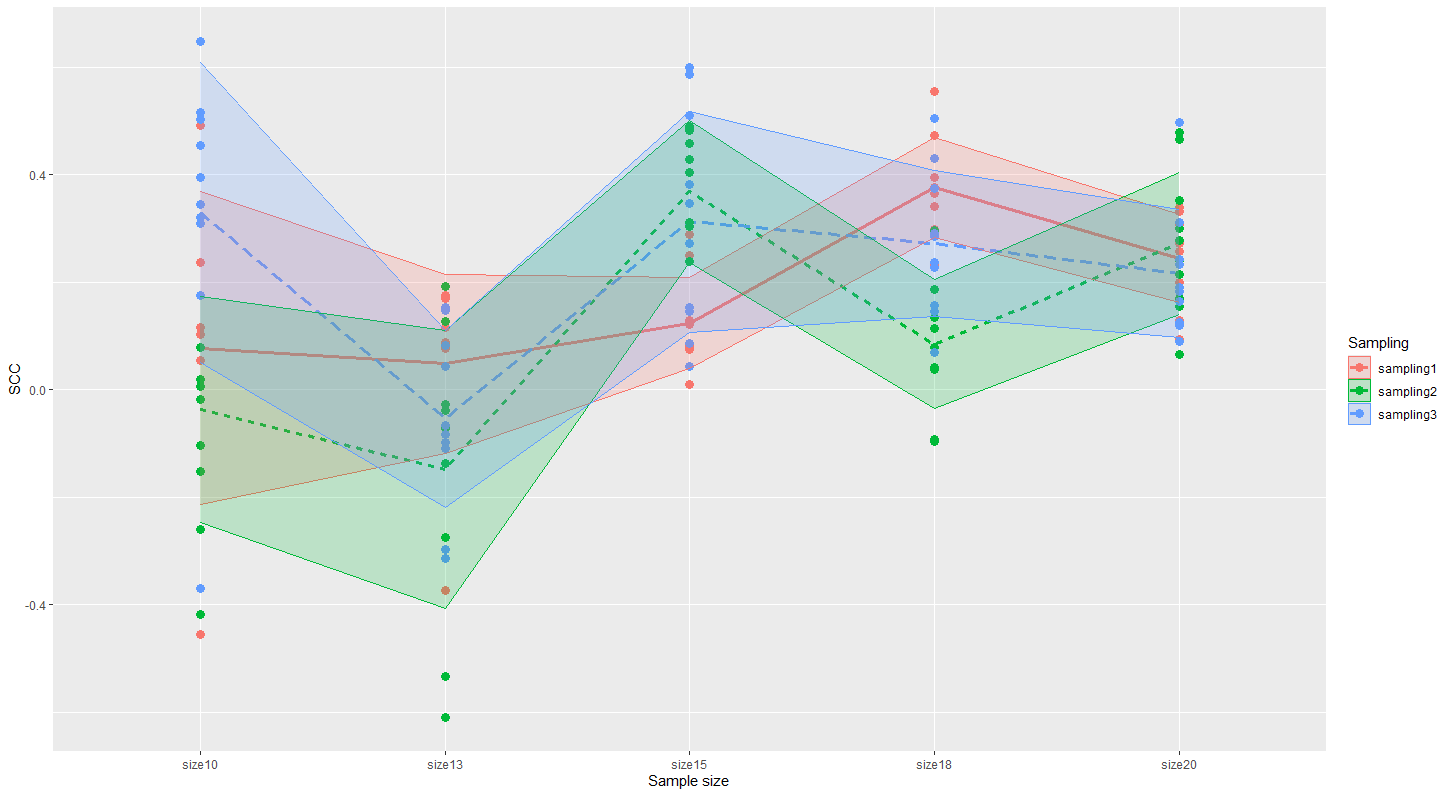


**Supplementary Table 1.** Genes involved in the six pathways and their corresponding weights based on two-step TL.

Please see Supplementary Table 1 in the Excel sheets.

**Supplementary Table 2**. An overview of the prediction model performances (in terms of Spearman Correlation Score) on datasets GDSC, HGCC, and GSE232173. The best performing model in each dataset is in bold. The p-values were calculated by Wilcoxon signed-rank test to compare the 10 SCC pairs, which were obtained from 10 repetitions of 5-fold/3-fold CV with identical partitions in both experiments.

|  | **Experiments** | **Results (mean ± sd)** |  |
| --- | --- | --- | --- |
| GDSC | #1a TMZ (without TL) | 0.085 ± 0.035 |  |
|  | #1b CPA (without TL) | 0.439 ± 0.019 |  |
|  | #1c BOR (without TL) | 0.363 ± 0.019 |  |
|  | #1d OXA (without TL) | **0.509 ± 0.015** |  |
|  | **Experiments** | **Results** | ***p*-values compared to the baseline Experiment #2** |
| HGCC | #2 (without TL) | 0.217 ± 0.052 |  |
|  | #3a TMZ (one-step TL) | 0.216 ± 0.057 | 0.846 |
|  | #3b CPA (one-step TL) | 0.208 ± 0.055 | 0.275 |
|  | #3c BOR (one-step TL) | 0.196 ± 0.044 | 0.193 |
|  | #3d OXA (one-step TL) | **0.244 ± 0.050** | 0.037 |
|  | **Experiments** | **Results** | ***p*-values compared to the best-performing model of Experiment #7** |
| GSE232173 | #4 (without TL) | 0.120 ± 0.118 | 0.010 |
|  | #5 (one-step TL) | 0.103 ± 0.126 | 0.006 |
|  | #6d (one-step TL) | 0.181 ± 0.078 | 0.014 |
|  | #7 (two-step TL) | **0.222 ± 0.079** |  |
|  | EN | -0.082 ± 0.192 | 0.002 |
|  | MGMT | 0.167 ± 0.148 | 0.275 |
|  | TS | -0.020 ± 0.266 | 0.014 |

**Supplementary Method.**

**(Hyper-)parameter tuning on OXA-based GDSC dataset.**

To start with, we used two hidden layers with the number of neurons (1000, 100); glorot is the default weight initializer; regularization kernel of 0.001, bias of 0.0001, the kernels after the input layer, first hidden layer, and second hidden layer were sigmoid, softplus, softplus. All the SCC scores reported below are the average of the 10 repetitions of the 5-fold cross validation.

Architecture:

| Activation function after each hidden layer (first, second, third) | Spearman correlation coefficients |
| --- | --- |
| **(sigmoid, softplus, softplus)** | **0.508** |
| (sigmoid, sigmoid, softplus) | 0.071 |
| (sigmoid, relu, softplus) | 0.506 |
| (relu, softplus, softplus) | 0.377 |
| (softplus, softplus, softplus) | 0.373 |

Then with the activation function (sigmoid, softplus, softplus):

| #hidden layers | #neurons in hidden layers (first, second, third) | Spearman correlation coefficients |
| --- | --- | --- |
| 1 | (500) | 0.506 |
| **2** | **(1000, 100)** | **0.508** |
| 2 | (1000, 10) | 0.501 |
| 2 | (500, 100) | 0.495 |
| 3 | (1000, 100, 10) | 0.503 |
| 3 | (1000, 500, 10) | 0.490 |

Regularization

| (kernel, bias) | Spearman correlation coefficients |
| --- | --- |
| (0.001,0.0001) | 0.508 |
| (0.0001,0.001) | 0.507 |
| **(0.0001,0.0001)** | **0.509** |
| (0.001,0.001) | 0.475 |

The initialization with He and RandomNormal normalization based on the optimal settings above:

| Initial normalization | Spearman correlation coefficients |
| --- | --- |
| He | 0.482 |
| RandomNormal | 0.494 |

**Computational resources used in the DL models.**

Below is the overview of the computational resources used when the DL model without TL and the DL model with two-step TL were run on the target set GSE232173 using a computer with Intel(R) Xeon(R) CPU E3-1270 V6 (4 Cores, 8 Logical Processors) and 64G RAM. Note that the DL model with two-step TL was pre-trained and therefore took less computational resource when it was applied.

|  | DL without TL | DL with two-step TL |
| --- | --- | --- |
| Execution time | 8-10 ms per sample per epoch | 5-6 ms per sample per epoch |
| Memory usage | 933.63 MB | 466.10 MB |
| Average CPU usage per epoch | 4.00 % | 2.75% |
